# Allergy or Tolerance: Reduced Inflammatory Cytokine Response and Concomitant IL-10 Production of Lymphocytes and Monocytes in Symptom-Free Titanium Dental Implant Patients

**DOI:** 10.1155/2013/539834

**Published:** 2013-09-11

**Authors:** Peter Thomas, Gerhard Iglhaut, Andreas Wollenberg, Dieter Cadosch, Burkhard Summer

**Affiliations:** ^1^Department of Dermatology and Allergology, Ludwig-Maximilians-University of Munich, Frauenlobstraße 9-11, 80337 Munich, Germany; ^2^Department of Dentistry, Georg-August University of Göttingen, Robert-Koch-Str. 40, 37075 Göttingen, Germany; ^3^Dental Surgery, Bahhnhofstraße 20, 87700 Memmingen, Germany; ^4^Clinic of Trauma Surgery, University Hospital of Zürich, Rämistr. 100, 8091 Zürich, Switzerland

## Abstract

Hypersensitivity reactions to titanium (Ti) are very rare. Thus, we assessed the proinflammatory response and also potential tolerance favoring *in vitro* reactivity of human blood lymphocytes and monocytes (PBMC) to Ti in healthy individuals (14 without, 6 with complication-free dental Ti implants). The proliferation index (SI) in lymphocyte transformation test (LTT) and production of cytokines linked to innate immune response (IL-1**β**, IL-6, and TNF**α**) or immune regulation (IL-10) were assessed in response to TiO_2_ particles or Ti discs. In both groups, the Ti-LTT reactivity was not enhanced (e.g., SI < 3). The control antigen tetanus toxoid (TT) gave adequate reactivity (median SI individuals without/with implant: 20.6 ± 5.97/19.58 ± 2.99). Individuals without implant showed higher cytokine response to Ti materials than individuals with symptom-free implants; for example, TiO_2_ rutile particle induced increase of IL-1**β** 70.27-fold/8.49-fold versus control medium culture. PBMC of 5 of the 6 individuals with complication-free Ti implants showed an *ex vivo* ongoing production of IL-10 (mean 4.18 ± 2.98 pg/mL)-but none of the 14 controls showed such IL-10 production. Thus *in vitro* IL-1**β**-, IL-6-, and TNF-**α** production reflects “normal” unspecific immune response to Ti. This might be reduced by production of tolerogenic IL-10 in individuals with symptom-free Ti dental implants.

## 1. Introduction

Cutaneous hyperreactivity to metals is common with nickel (Ni) and to a lesser extent cobalt (Co) and chromium (Cr) being typical elicitors. The underlying mechanism is a T-lymphocyte driven antigen-specific delayed type hypersensitivity [1]. The general population has repeated contact to titanium (Ti) in the form of TiO_2_ in paints and whitening agents, in many sunscreens and skin care products. In the industrial field, many workplaces include the handling of Ti, as it is used in metallurgic, pharmaceutical, and food industries. Furthermore, Ti-based materials are widely used in orthopaedic surgery and dental implantology, as they offer a fast osseointegration. However, only very few case reports or small case series exist describing potential hypersensitivity reactions to Ti, for example, axillary dermatitis to a Ti lactate containing antitranspirant; local eczema or inflammatory granulomatous reactions to Ti-based pacemakers; impaired wound/fracture healing upon osteosynthesis; and loosening of hip arthroplasty in association with peri-implant Ti wear particles and patients showing skin test reactivity to a Ti containing ointment [2–6]. On the other hand, patients prior to implantation or already having Ti-based implants may be concerned by reports on the risk of local or systemic adverse health effects. Some authors have reported high frequency of “hyperreactors” against Ti using lymphocyte transformation test (LTT) [7]. However, there is a controversial discussion regarding Ti related immune reactivity [8, 9] since the LTT may at the most indicate sensitization leaving its clinical significance open [10–12]. Furthermore, LTT has so far been accepted for and is restricted to cases of suspected Ni, beryllium, or drug hypersensitivity [10]. 

Although Ti has been regarded as “biologically inert,” more recent research has shown that Ti-based implants undergo biocorrosion and release metal ions and particles into surrounding tissues. Various mechanisms, including mechanical wear—particularly in arthroplasty prostheses—and electrochemical corrosion, may account for such local metal release and potential dissemination [13–15]. It seems that systemic dissemination of Ti ions is not limited to hip arthroplasty but may encompass also patients with osteosynthesis (e.g., intramedullary nails) [16–18].

The formation of periprosthetic osteolytic lesions is a major clinical problem in joint replacement. The innate immunity has been implicated to be the predominate responding mechanism to wear particles, and, correspondingly, TNF*α*, IL-6, and especially IL-1*β* expression has been found in areas surrounding failed arthroplasty [13, 19, 20]. 

In addition to soluble and particulate materials derived from metal implants made of CoCrMo alloy [21], Ti wear particles have also been shown to induce IL-1*β* secretion through activation of the inflammasome complex [22]. 

A similar danger signal-related release of mediators like IL-1*β* or IL-6 has also been observed in Ti dental implant patients with peri-implantitis [23].

Assuming that Ti is released from implants, the next question would be to quantify the “normal” inflammatory mediator production in response to it. To answer this question, our investigations addressed two aims. The first aim is, to assess the inflammatory mediator production and LTT reactivity of human lymphocytes and monocytes collected from healthy individuals and exposed to Ti particles and discs *in vitro*. Thus, the “normal” range of such response in individuals without implant should be established. The second aim is, to investigate potential variations of this response in healthy individuals with symptom-free Ti dental implants.

## 2. Materials and Methods

### 2.1. Titanium Materials

**Figure 1:**
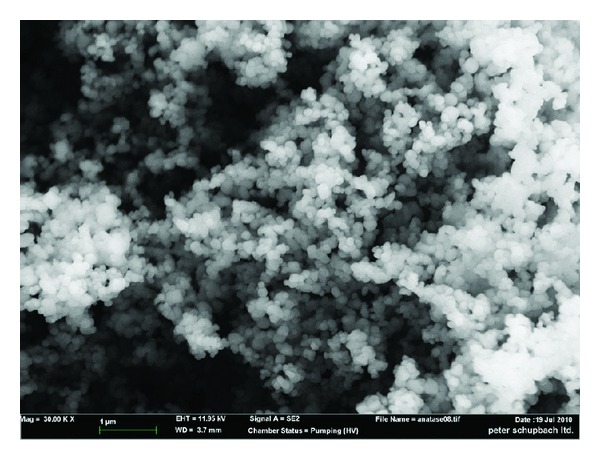
Ti-particles in anatase structure, mostly agglomerated.

Since metallic Ti spontaneously oxidises to titanium dioxide in air or water and forms a protective surface layer against further oxidation, commercially pure titanium dioxide (TiO_2_) particles (Sigma, Deisenhofen, Germany) were obtained having anatase or rutile as the main crystallographic structure: the anatase particles had a size <44 *μ*m (product no. T8141) and the rutile particles a size <5 *μ*m (product no. 224227). Particles of these two different size ranges were chosen according to their potential of different inflammasome complex activation (e.g., “frustrated phagocytosis” generated by large-sized particles or “endosomal destabilization” by readily phagocytized smaller particles) [24, 25]. After having been autoclaved, the particles were suspended in sterile cell culture medium for subsequent studies. The anatase particles tended to agglomerate in the cell culture medium (Figure 1). In addition to TiO_2_ particles, Ti discs were used. They consisted of commercially available pure Ti (grade 4) and were supplied by Nobel Biocare with a diameter of 6 mm and a thickness of 1.75 mm. Three surface modifications of the discs were applied: as-machined (MA), spark anodized (SA), and sand blasted and acid etched (SBA). The surface roughness was measured with a white light interferometer and amounted 0.5 *μ*m for the machined surface, 1.25 *μ*m for the spark anodized surface, and 1.75 *μ*m for the sand blasted and etched surface modifications.

The discs were cleaned and sterilized by gamma irradiation. They were sent to us in 96% ethanol. Before using them for cell culture experiments, the discs were removed from the ethanol and air-dried in a sterile environment.

### 2.2. Blood Donors

A total of 20 healthy nonsmoking (12 m, 8 f; 21–62 years) volunteers were recruited for this project among the staff of the University Clinic to minimize potential confounding factors. All participants were healthy as assessed by regular occupational medicine checkup including examination of systemic infectious diseases; there was no uptake of immunomodulating/immunosuppressive medication; there was no previous or ongoing immunomodulatory, for example, hyposensitization immunotherapy; in the two individuals with atopy, blood sampling was done in the pollen free season. All individuals were nonsmokers. The study was approved by the local ethics committee. Six of the individuals had symptom-free Ti dental implants; for example, there were no complaints and also no signs of inflammation upon dentist's check. Fourteen did not have dental implants. An overview of the participant characteristics is given in Table 1. For the *in vitro* experiments, 60 mL of heparinised blood was taken by peripheral venous puncture. 

### 2.3. Cell Preparation and Stimulation

Peripheral blood mononuclear cells (PBMC) were isolated from the freshly drawn blood samples by Ficoll-Paque (Pharmacia, Uppsala, Sweden) density gradient centrifugation. The PBMC were then resuspended at 1 × 10^6^/mL in supplemented culture medium (Biochrome, Berlin, Germany) containing autologous serum and were stimulated in quadruplicate culture over 6 days under the following conditions: culture medium alone (negative control), of T-cell mitogen phytohemagglutinin (PHA, Biochrom, Berlin, Germany) 2.4 *μ*g/mL or the recall antigen tetanus toxoid (TT, Chiron Behring, Berlin, Germany) 5 *μ*g/mL; addition of TiO_2_ as TiO_2_ particles (anatase structure and rutile structure, 10^−4 ^M, 10^−5 ^M, Sigma, Germany); and layering over titanium discs (3 variants, for example, MA, SA, and SBA). One set of cultures was used to obtain the conditioned supernatants. Here, instead of PHA the pan T cell cytokine stimulator phorbol-myristate-acetate (PMA, Sigma, Munich, Germany; 150 mmol) was added. The other set was used for the proliferation assay. 

### 2.4. Lymphocyte Proliferation Assay

To assess the proliferative response of the PBMC cultured with Ti discs or TiO_2_ particles, cells were pulsed with 3H-thymidine for the last 18 h after 5 days of culture (Amersham Biosciences, Freiburg, Germany). In order to determine the incorporated radioactivity, labelled cells were harvested, and a scintillation counter was used. The proliferative response was expressed as stimulation index (SI), which was calculated by the ratio of mean counts per minute (cpm) of stimulated to unstimulated cultures. A SI > 3 was regarded as positive [26]. 

### 2.5. Analysis of Cytokine Production

After the 6-day culture, supernatants of the nonradioactive culture (pooled from each of the duplicate experiments) were assayed for the presence of pro-inflammatory cytokines mostly linked to innate immune response, for example, IL-1*β*, IL-6, and TNF-*α*, and for the antiinflammatory cytokine IL-10. Levels of these cytokines were determined by use of fluorescent antibody marked microparticles in a cytometric bead assay (BD Biosciences, Heidelberg, Germany) and flow cytometry. In order to take account of potential spontaneous or preexisting cytokine liberation, the cytokine levels found upon stimulation were put into relation to the baseline cytokine amount determined in the culture with medium only (control without stimuli). This relation is given as fold increase. 

### 2.6. Statistical Analysis

All calculations for data assessment were performed using SPSS software. For comparison of the 2 groups the Wilcoxon rank-sum test was used. *P* < 0.05 was considered significant. Data are presented by box plot indicating the 25th and 75th percentiles and the median. The frequency of spontaneous IL-10 production was evaluated by the use of the Fishers exact test.

## 3. Results

### 3.1. Proliferative Response in the LTT

**Figure 2:**
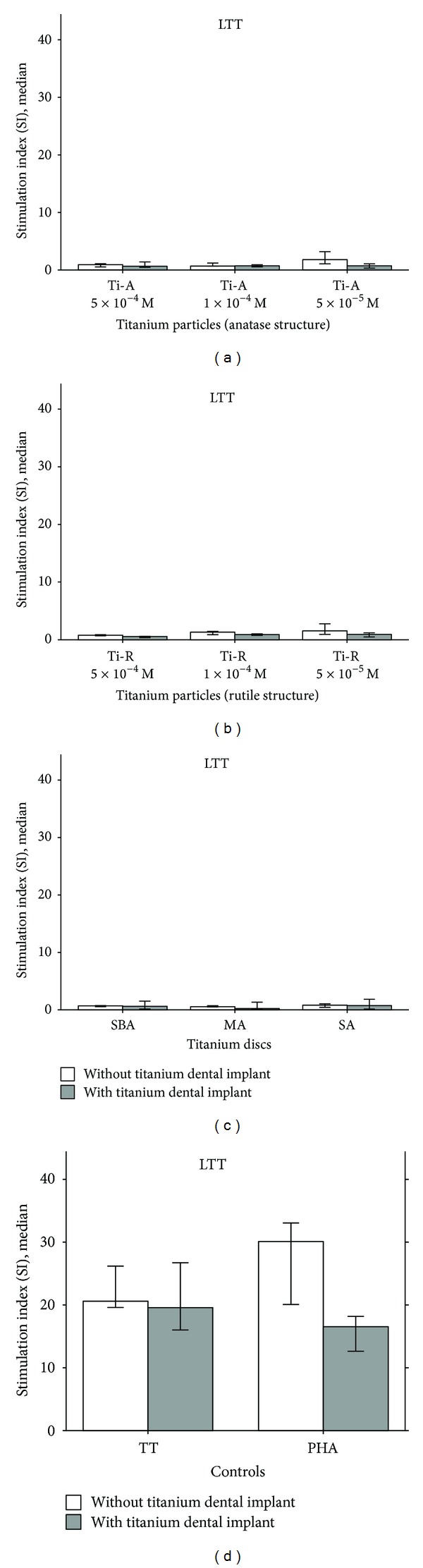
Lymphocyte proliferation response of the PBMC of the 14 healthy individuals without titanium dental implants (open bar) and 6 blood donors with symptom-free titanium dental implants (closed bar). Reactivity to stimulation with titanium particles in rutile and anatase structures (5 × 10^−4 ^M, 1 × 10^−4 ^M, 5 × 10^−5 ^M) ((a) and (b)), the three titanium metal discs (SBA, MA, and SA) (c) and to the controls (recall antigen) tetanus toxoid (TT) and (pan T cell mitogen) phytohemagglutinin (PHA), over 6 days. Stimulation index (SI) is given as ratio of stimulated culture to unstimulated culture.

As expected, increased proliferation was seen to the T-cell stimulator PHA in both groups (median SI in implant patients: 16.5 ± 6.24; median SI in healthy controls without implant: 30.1 ± 5.66) reflecting the absence of immunosuppressive influences and showing adequate T-cell response. To assure that cells would properly react to a protein recall antigen, we used tetanus toxoid (TT) based on everybody's previous vaccination-dependent exposure to it. Accordingly, stimulation with TT was able to induce a (specific) T-cellular proliferative response with median SI of 20.6 ± 5.97 for healthy control subjects and 19.58 ± 2.99 for individuals with symptom-free Ti dental implants. These data reflect a valid immunisation status to TT in the individuals tested and a corresponding adequate *in vitro* response. When assessing the LTT response to the different Ti particles (rutile and anatase) and to the Ti discs, the median SI always remained below the level of 3. Thus, there was no enhanced T-lymphocytic proliferation in both the individuals without and those with symptom-free Ti dental implants. The respective data are summarized in Figures 2(a)–2(d).

### 3.2. Cytokine Production

**Figure 3:**
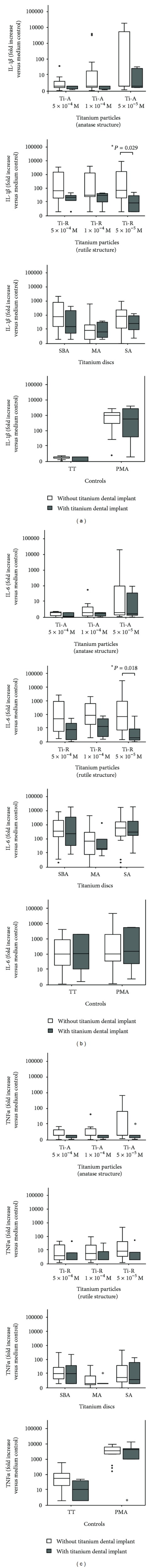
Production of IL-1*β* (a), IL-6 (b), and TNF*α* (c) of PBMC of the 20 blood donors (14 healthy individuals without titanium dental implants = open bar and 6 with symptom-free titanium dental implants = closed bar) after stimulation with titanium particles in anatase and rutile structures (5 × 10^−4 ^M, 1 × 10^−4 ^M, 5 × 10^−5 ^M) and the three metal discs over 6 days. Fold increase versus stimulation with medium alone is given.

To determine whether *in vitro* exposure to Ti particles or Ti metal discs was inducing cytokines of an innate immune response, the levels of IL-1*β*, IL-6, and TNF-*α* were assessed. The respective results are given in Figures 3(a)–3(c). Whereas the pan stimulator PMA leads to high, comparable levels of IL-1*β*, IL-6, and TNF-*α* in both groups, there was—to a varying extent—a reduced release of those cytokines from Ti exposed PBMC of individuals with symptom-free Ti dental implants. For example, for TiO_2_ particles (rutile structure 5 × 10^−5 ^M), this difference was for IL-1*β*  70.27 ± 672.31 median fold increase (patients without implants) versus 8.49 ± 7.92 median fold increase (patients with implant; *P* < 0.05). With regard to particles in general, rutile structure seemed to induce higher response, which could be related to the smaller size and the higher surface-to-volume ratio of these particles compared to the anatase particles. Within the three Ti disc variants, “MA” gave a lower response of these three cytokines, which could be due to the smaller surface area of the machined discs compared to the “SA” or “SBA” discs. With regard to TT, its specific recognition did not trigger danger signal (inflammasome) related IL-1*β* release. 

**Figure 4:**
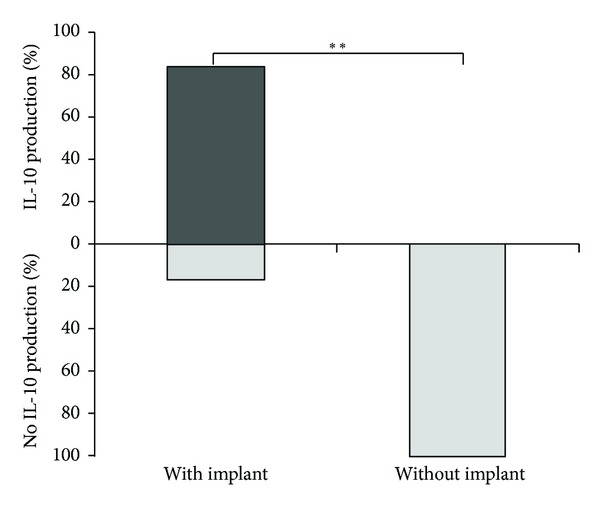
Frequency (%) of spontaneous IL-10 production of PBMC of the blood donors (6 with, 14 without dental Ti implant) after 6 day culture with medium alone. **P < 0.001 (Fishers exact test).

To show whether there was some modulation of (monocytic) cytokine response to both Ti particles and disc surfaces by the presence of autologous T-cells, *ex vivo* PBMC cultures were used. Furthermore, we compared PBMC of healthy blood donors without exposure to Ti dental implants to those of symptom free carriers of Ti dental implants. Since IL-10 plays a crucial role in downregulation of inflammatory responses, its expression was monitored. In the unstimulated cultures IL-10 was only produced by PBMC derived from symptom-free carriers of Ti dental implants. None of the 14 individuals without implant, but 5 of the 6 implant carriers showed such basal IL-10 production (Table 2 and Figure 4). These data indicate that there were already *in vivo* IL-10 generating, tolerance favoring cells in those individuals.

## 4. Discussion

Released Ti may be detected not only in tissues surrounding joint replacement, but also in regional lymph nodes in association with Ti screws or mini plates [15, 27–29]. Ti is also encountered in tissues adjacent to dental Ti implants, although not as extensive—and fretting maybe one of the underlying mechanisms [30]. The wear particle related macrophage activation stimulates the release of proinflammatory cytokines such as TNF-*α*, IL-6, prostaglandin E2, and IL-1*β* [31, 32]. These cytokines—particularly IL-1*β*—are considered to directly or indirectly mediate osteoclastic differentiation and activation and ultimately bone resorption [33]. The role of Ti in the induction of a IL-1-related peri-implant inflammation with subsequent bone resorption has been shown in animal (mice) models using intramedullary Ti rod implantation and Ti particles introduced onto the calvarium [20, 34].

In analogy to orthopaedic implants, many reasons and risk factors for dental implant failure have been published, including oral bacterial load, smoking, presence of diabetes mellitus, or iatrogenic factors [35–38]. In contrast, the role of hypersensitivity or proinflammatory cytokine response to Ti is still a controversial issue [4, 9]. There are no evaluated patch test preparations for skin testing in case of suspected Ti hypersensitivity. In addition the sometimes used TiO_2_ preparation may produce unspecific reactions and false positive patch test reactions due to impurities [39]. This might also be considered as differential etiology in intolerance reactions to Ti materials [40]. Thus, the LTT is sometimes used to look for Ti sensitivity. The LTT is used for measurement of proliferation in PBMC cultures in response to addition of antigens, to which sensitization has occurred. However, in the present study, a significant increase of SI response to TiO_2_ particles or Ti-discs was not observed. This is in accordance with experiments of Park et al. showing that, in the local lymph node assay the SI value did not increase to TiO_2_ particles, but to eugenol as positive control [41]. However it has been shown that mouse and human macrophages respond to Ti particles with the production of IL-1*β* [9, 22]. This IL-1*β*-associated response to Ti particles is associated with activation of the NALP3 inflammasome. A similar cytokine response had been already linked to inflammasome activation in the case of *in vitro* response to silica crystals and aluminium salts [42]. In the murine model, Ti particle exposure leads to IL-1*β* mediated proinflammatory responses including neutrophil recruitment and bone resorption [34]. In our study, we hypothesized that proinflammatory mediator release, namely, IL-1*β*, IL-6, and TNF*α*, would reflect a “normal” unspecific response in patients exposed to Ti particles. We used PBMC cultures to examine whether such mediator release could be modulated by the presence of autologous T-lymphocytes. 

Our results show that coculture of human PBMC with either Ti particles or Ti discs leads to release of considerable amounts of IL-1*β*, IL-6, and TNF-*α*. When using a typical recall antigen (tetanus toxoid) such secretion of IL-1*β* was not seen. However, a reduced release of cytokines by PBMC was observed in patients with symptom-free Ti dental implants. We thus wondered if, by using PBMC, that is, in the presence of T-lymphocytes, some regulatory mechanisms may be demonstrable. When considering scenarios with production of systemic proinflammatory mediators, for example, early after surgery, cardiac arrest, or in sepsis patients, anti-inflammatory mediators—namely IL-10 or IL-1*β*Ra—have been concomitantly found [43–45]. Martire-Greco and coworkers investigated the effect of the presence of IL-10 in the cultures of human neutrophils (PMN) subsequently exposed to LPS and found that the TNFa and IL-8 secretion was markedly attenuated upon addition of IL-10 to the cultures [46]. With regard to our experiments, in contrast to PBMC of the 14 individuals not yet exposed to Ti, those of individuals with well tolerated Ti dental implants produced the potent anti-inflammatory cytokine IL-10 *in vitro*. Five of the 6 implant carriers showed PBMC with the basal production of IL-10. As reported by Pinke et al., in their investigation of unstimulated PBMC cultures from healthy young individuals (20 to 50 years old), the large majority showed no basal IL-10 production *in vitro* [47]. Thus, our observation seems to be a relevant finding, as IL-10 has an important role in the *in vivo* regulation in sense of a negative feedback loop reducing the release of inflammatory mediators and dampening an acute inflammatory response [48–50]. There is, for example, also an increased expression of IL-10 by PBMC of pregnant individuals with a probable role maintaining fetal tolerance [51, 52]. IL-10 is also induced in circulating PBMC upon specific immunotherapy with pollen allergens [53]. In general, many cells of the innate and adaptive immune system may produce IL-10, and even do so together with proinflammatory cytokines [54]. A recent paper has suggested, that, by understanding the regulatory mechanisms for IL-10 expression, this cytokine could be a therapeutic target to reduce inflammatory tissue damage [54]. As IL-10 was found to be secreted from the *ex vivo* obtained PBMC of individuals with symptom-free Ti implants, it is tempting to speculate that IL-10 prevent from peri-implant inflammation, even though peri-implant inflammatory cytokine production may also be present in healthy subjects without implant failure. Correspondingly, Nowzari et al. concluded from their experiments that there is an inevitable and ongoing spectrum of microorganisms and interleukins around teeth and implants [55]. There is, however, still controversy about the interplay and significance of the different interleukins in crevicular fluid and peri-implantitis [23]. At present, we know little about the immune mechanisms leading to well-tolerated Ti dental implants, but our results indicate that adaptive mechanisms may exist. Future studies will determine which factors and cell populations will add to this protective tolerance. 

## Figures and Tables

**Table 1:** Characteristics of the blood donors.

Initials	Gender	Age	Ti dental implant		Other dental materials	CMI	Patch test	Smoker	Atopy
			Number	Since					
H.T.	m	45	1^a^	2006	Crown; ceramic; metal	No	n.d.	No	No
T.B.	m	21	3^b^	2007	No	No	n.d.	No	No
K.C.	m	41	1^c^	2003	Crown; resin; ceramic	No	n.d.	No	ra
K.H.	m	49	1^d^	2009	Crown; ceramic; metal	No	n.d.	No	ra
F.M.	m	56	1^e^	2006	Crown; ceramic; metal	No	Negative	No	No
S.R.	f	62	6^f^	2005	Crown; ceramic	No	n.d.	No	No
S.L.	f	25	None		Resin; ceramic	Yes	Ni; Lan.	No	No
C.S.	m	23	None		No	No	Negative	No	ra; aa; ae
H.C.	f	23	None		No	No	Negative	No	No
P.M.	f	24	None		No	No	Negative	No	No
G.G.	m	31	None		No	No	n.d.	No	No
S.J.	m	34	None		Crown; resin; ceramic	No	Thiomer; Merc.	No	No
F.T.	f	29	None		No	No	n.d.	No	ra
A.A.	m	34	None		Ceramic; amalgame	No	n.d.	No	ra
F.K.	m	46	None		Crown; metal	No	n.d.	No	ra
N.J.	m	33	None		Crown; resin	No	n.d.	No	No
S.F.	m	23	None		Crown; metal	No	n.d.	No	No
E.R.	f	30	None		No	No	Negative	No	ra; ae; aa
B.P.	f	43	None		Crown; resin; ceramic; metal	No	Negative	No	No
R.C.	f	35	None		Crown; ceramic	No	n.d.	No	No

CMI: cutaneous metal intolerance; aa: allergic asthma; ra: rhinoconjunctivitis allergica; ae: atopic eczema.

Patch test reaction: Ni: nickel; Lan: lanolin; Thiomer: thiomersal; Merc: mercury-II-amidchloride; negative: no test reaction; n.d.: not done.

^a,b,c,d,e,f^Implant devices of different manufacturers (details known to the authors) made of pure titanium or TiAlVa alloy.

**Table 2:** Spontaneous cytokine production by the *ex vivo* obtained PBMC of the 20 individuals. Cytokine levels were assessed in supernatants of unstimulated (e.g., only culture medium) 6 d cultures.

	Individuals with titanium dental implant (*n* = 6)		Individuals without titanium dental implant (*n* = 14)	
	Mean (pg/mL)	±	Mean (pg/mL)	±
IL-1*β*	9.04	16.87	23.39	65.47
IL-6	688.33	1566.10	1909.8	4958.15
IL-10	4.18	2.98	0	0
TNF*α*	1.02	1.93	1.75	4.52
